# TOR inhibition interrupts the metabolic homeostasis by shifting the carbon–nitrogen balance in *Chlamydomonas reinhardtii*

**DOI:** 10.1080/15592324.2019.1670595

**Published:** 2019-10-04

**Authors:** Umarah Mubeen, Patrick Giavalisco, Camila Caldana

**Affiliations:** a Molecular Physiology Department, Max Planck Institute of Molecular Plant Physiology, Potsdam-Golm, Germany; b Max Planck Institute for Biology of Ageing, Cologne, Germany

**Keywords:** Target of rapamycin kinase, growth, metabolism, *Chlamydomonas reinhardtii*

## Abstract

The allocation of nutrient resources to growth and metabolism is an essential function for controlling biomass accumulation in photoautotrophic organisms. One essential protein complex involved in this process is the target of rapamycin (TOR) kinase. It has been shown that the inhibition of TOR leads to a considerable upsurge in the amino acid levels. This molecular phenotype relies mainly on the availability of light, carbon (C) and nitrogen (N). To validate the time-resolved response of C and N metabolites, we used a targeted gas chromatography mass spectrometery (GC-MS)-based metabolomic approach, where we examined the response of *Chlamydomonas reinhardtii* upon TOR inhibition under C-limited condition, namely extended darkness. Contrary to C-supplemented conditions, the rapid increase in the amino acid levels is suppressed almost completely 4 h after TOR inhibition, confirming that C supply is essential to raise the amino acid levels mediated by their *de novo* synthesis. An exception to this observation was the levels of aspartate, which is presumably synthesized via the anaplerotic pathway. In agreement with previous reports, TOR repression, under these C-limited conditions, leads to a significant reduction in the C/N ratio, corroborating with the crucial role of the pathway in maintaining the metabolic balance of the cells and consequently propelling growth.

The regulation of metabolic homeostasis is critical to biosynthetic processes and cellular functions. Target of rapamycin complex 1 (TORC1) – an essential multiprotein kinase – is known to integrate a wide range of extracellular and intracellular signals to regulate cell growth and metabolism.^1^ In past years, a number of studies have investigated the impact of TOR inhibition on the metabolism of the photosynthetic unicellular green algae *Chlamydomonas reinhardtii* under diverse growth conditions, including mixotrophic (photo-heterotrophic) and autotrophic (photoautotrophic) growth.^2–6^ The analysis of the photoautotrophic cultures throughout a diel (24 h) time course revealed key metabolic signatures, marked by the massive accumulation of several storage compounds, such as starch, triacylglycerols, and amino acids, upon TOR inhibition.^3,5^ While the coordinated and significant accumulation of starch and triacylglycerols occurred approximately 1–2 h after TOR inhibition, the escalation in the amino acid levels was noticed as early as 15 min after the TOR inhibitor rapamycin was administered.^5^ This metabolic remodeling occurred mainly due to a shift from anabolic to catabolic metabolite utilization. Particularly, the striking accumulation of amino acids was mainly attributed to the repression of translation and/or the induction of autophagy^7,8^ mediated by TOR inhibition in this organism.^9,10^

More recently, in contrast to previous assumptions, the main process leading to the rapid accumulation of almost all amino acids has been attributed to the significant and immediate induction of their *de novo* synthesis in *Chlamydomonas*.^11^ By combining stable isotope labeling experiments using ^13^C acetate and ^15^N ammonia, metabolic profiling, and enzymatic activity assays of central nitrogen-assimilating enzymes, it has been shown that the inhibition of TOR triggers immediate nitrogen uptake and assimilation. This process occurs by consuming carbon (C) substrates from the central metabolism to sustain high levels of amino acids,^11^ presumably to enable TOR re-activation, and therefore, maintaining the metabolic homeostasis. This assumption was supported by the previous experiment, in which the rapamycin treatment was applied in synchronized *Chlamydomonas* CC-1690 cells kept for 11 h in darkness.^12^ Even though the levels of all measured amino acids immediately increased after 0.1 h of drug administration, their levels could not be sustained at higher concentrations over time, leading to a drop after 0.5 h of the treatment. These results indicated that the higher levels of amino acids after TOR inhibition are depending on C availability. In agreement with this hypothesis, the response of the amino acids after rapamycin treatment is even more restricted when the cells were exposed to a more drastic nutrient limitation, namely low C and low nitrogen levels. Surprisingly, it was shown that this response is not conserved for all amino acids. Indeed, the levels of glutamate, glutamine, aspartate, asparagine, alanine, serine, threonine, glycine, and proline stayed significantly elevated after 1 h of rapamycin treatment, indicating that there might be an autonomous cellular mechanism to keep them high under these conditions.^11^

As the accumulation of free amino acids due to the induction of *de-novo* synthesis after TOR repression relies on the availability of nutrient resources,^11^ it was expected that by extending the dark phase, C reserves will be exhausted even more, thus preventing the high amino acid content phenotype. Hence, the present study was aimed to evaluate how the extended depletion of C reserves would influence the levels of amino acids. For this purpose, synchronized cultures of *C. reinhardtii* wild-type strain CC1690 (mt+; Sager 21 gr)^12^ were treated either with dimethyl sulfoxide (DMSO) (Control) or with 5 µM rapamycin in DMSO 1 h before the end of the dark phase (11th hour in dark phase of 12 h/12 h light/dark cycle) and exposed for 3 h of extended darkness. The sampling was done at 0.1, 0.25, 0.5, 1, 2, and 4 h after the treatment in five replicates of ~30 million cells per sample. Primary polar metabolites, including the amino- and organic acids, were extracted and analyzed by GC-MS, as described in detail previously.^12^

A critical indicator of the metabolic balance of the cell is the ratio of carbon and nitrogen (C/N), which can be assessed by the ratio of conjugated amino acid/keto acid pairs. Here, especially, the pair 2-oxoglutarate (2-OG) to glutamate was shown to be particularly indicative.^13^ Accordingly, we first re-examined the behavior of the C/N ratio in our previously published work. Under control conditions, the ratio oscillates along the 24-h diel cycle,^5^ corresponding to different growth phases of the cell cycle (Figure 1a). Interestingly, the cells treated with rapamycin not only displayed a reduced C/N ratio in comparison to the control but also abolished the oscillatory pattern (Figure 1a), marking a detachment of this pattern from the growth phases. Similarly, the short-term TOR inhibition under different growth conditions (i.e. presence of light, C limitation, and both C and N limitation) leads to a reduction of C/N ratio (Figure 1b–d). It is also important to note that the different growth conditions also influence the C/N ratio as indicated by the controls of different experiments conducted under diverse nutrient regimes (Figure 1a–e). This highlights the changes in the C/N balance due to the availability of light and nutrients. Nevertheless, the addition of rapamycin to the cultures led to a further reduction in this ratio compared to the controls, regardless of the nutrient regime, suggesting that the shift in the C/N balance is one of the primary causes of growth reduction upon TOR inactivation.

**Figure 1.**
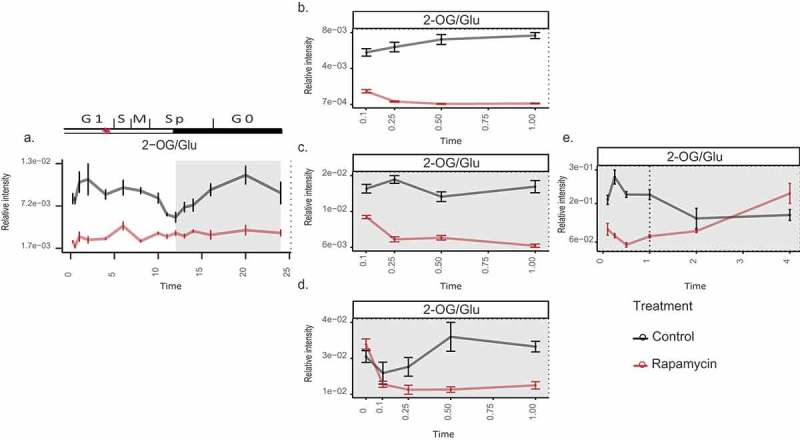
Changes in the C to N ratio (derived from 2-OG/Glu) upon TOR inhibition in *Chlamydomonas*. (a) Over 24-h cell cycle (the red-star symbol in the plot marks the commitment point (CP) of the cell cycle, where the cells grow large enough to commit to divide); (b) in the presence of light (batch culture); (c) dark; (d) dark and no nitrogen; and (e) extended dark*ness* (dotted line indicates the start of extended dark phase). Sampling time is given on the x-axes, while relative intensities are given on the y-axes. Note that 0 h sampling was carried out only in (d) to account for the error introduced due to the nitrogen starvation, before applying rapamycin treatment.^11^ The metabolites were measured using GC-MS platform. Samples are represented as the mean of six replicates ± SE (a) and five replicates ± SE (b–e). Significance testing was performed by ANOVA2. The data plotted in (a–d) were retrieved from previously published works.^11^

Although this trend was also observed initially in the extended darkness experiment (Figure 1e), the differences in the C/N ratio of the control- and rapamycin-treated cultures were minimized over time. Although the amino acid levels exhibited rapid upshift upon TOR inhibition,^5,11^ the limited availability of the C reserves restricted the elevated amino acid phenotype leading to an insignificant difference in the C/N ratios under long-term darkness (Supplementary Table 1). Notably, the swift and substantial upshift in the amino acid levels in comparison to the respective carbon substrates are anticipated for the reduced C/N ratio upon TOR inhibition.

Furthermore, in line with the previously reported data after TOR inhibition under nutrient limitation,^11^ the levels of almost all amino acids were reduced in the rapamycin-treated samples when compared to their corresponding control under the extended dark treatment. The only exceptions were the amino acids aspartate (Asp) and glutamine (Gln) that surprisingly still maintained their levels high by the end of extended darkness under TOR inhibition (Figure 2). Notably, though both amino acids exhibited a similar trend, only the Asp increase was significant (*p* < .05) (Supplementary Tables 1 and 2). The synthesis of Asp is catalyzed by aspartate aminotransferase through a reversible reaction using the tri-carboxylic acid cycle (TCA) cycle intermediate oxaloacetate (OAA) and glutamate as the C and amino substrates, respectively. The direction of the reaction depends on the concentration of the precursors. Although the level of 2-OG was previously shown to increase after TOR inhibition,^11^ the level of OAA was not reported due to the chemical instability of this metabolite. One of the routes for the generation of OAA is through anaplerotic reactions, where pyruvate is carboxylated via pyruvate carboxylase.^14^ The significantly high levels of Asp observed at the end of extended darkness indicated that this anaplerotic reaction is coupled with the reversible cataplerotic reaction by aspartate aminotransferase, thus potentially sustaining the high levels of Asp even after long-extended darkness. Additionally, the levels of OAA precursors, such as malate, had been shown to be reduced after TOR inhibition.^11^ Apart from OAA substrate, malate is also utilized for pyruvate production, which can be another substrate for amino acid (Ala) synthesis. Hence, it is hypothesized that the flux of the C substrates to different amino acids is strictly regulated upon TOR inhibition, which could be proven by assessing the activities of enzymes involved in various anaplerotic and cataplerotic reactions under these conditions.

**Figure 2.**
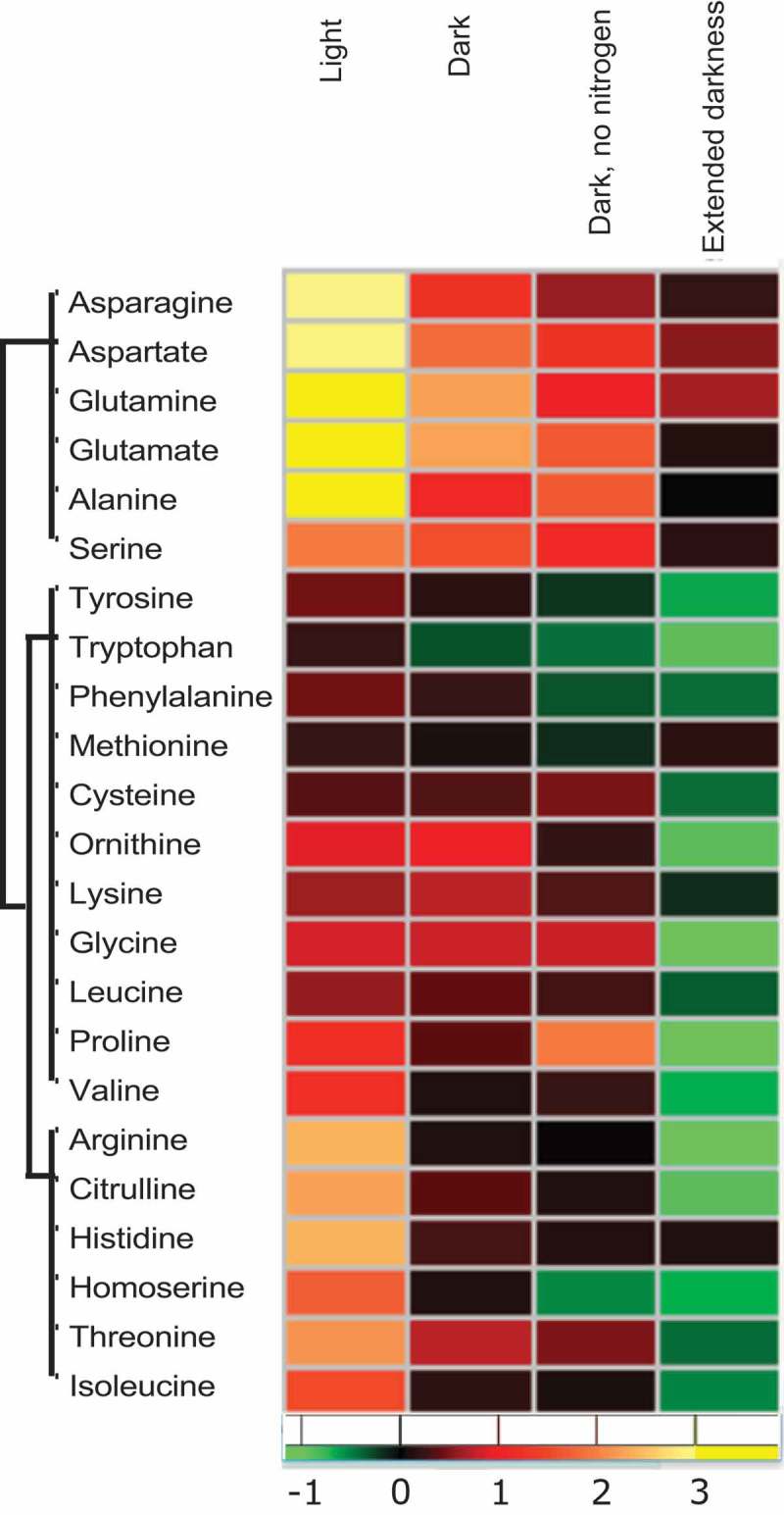
Changes in amino acid levels depend on the resource availability. Heatmap showing fold change (Log _2_) in the amino acid levels after 1 h of treatment with 5 µM rapamycin or equal concentration of drug vehicle (in light, dark, and dark and no nitrogen experiments)^10^ and after 4 h of the same treatment in extended dark experiment. Fold changes were calculated by dividing the mean of rapamycin samples (five replicates) with the mean of control samples (five replicates). The data for the first three experiments are obtained from the study by Perez-Perez et al.,^10^ while the extended darkness experiment is conducted in the present study.

In lieu of these observations, we hypothesize that the stored C reserves are potentially utilized to fuel required C substrates for maintaining the high amino acid levels. However, a prolonged dark period potentially exhausts the C reserves; thus, amino acid levels in rapamycin-treated cultures drop down to control or even below control. Importantly, it would be useful to evaluate the contribution of autophagy that is triggered upon TOR inhibition to refuel the emptied storage reserves for *de-novo* amino acid synthesis. Contrary to the other amino acids, the sustained levels of Asp potentially indicate the anaplerotic flux of pyruvate to OAA to drive the synthesis of Asp. Taken together, the present study highlights the significance of the TOR pathway in maintaining metabolic homeostasis for biosynthetic growth of the cells.
