# Supplementary material for: TOR inhibition interrupts the metabolic homeostasis by shifting the carbon–nitrogen balance in Chlamydomonas reinhardtii
Source: Plant Signal Behav. 2019 Oct 4;14(11):1670595. doi: 10.1080/15592324.2019.1670595 (PMC6804693; doi:10.1080/15592324.2019.1670595)
Supplement: Supplemental Material [file kpsb-14-11-1670595-s001.docx]

**Supplementary Material**

**Supplementary Table 1**. Relative intensities of key metabolites and ratio of 2-OG/Glu under extended darkness

**Supplementary Table 2**. Relative intensities of key metabolites after TOR inhibition under different growth regimes.
